# 2,2-Diphenyl-*N*-(1,3-thia­zol-2-yl)acetamide

**DOI:** 10.1107/S1600536812013840

**Published:** 2012-04-06

**Authors:** Hoong-Kun Fun, Chin Wei Ooi, Prakash S. Nayak, B. Narayana, B. K. Sarojini

**Affiliations:** a X-ray Crystallography Unit, School of Physics, Universiti Sains Malaysia, 11800 USM, Penang, Malaysia; b Department of Studies in Chemistry, Mangalore University, Mangalagangotri 574 199, India; c Department of Chemistry, P. A. College of Engineering, Nadupadavu, Montepadavu, PO, Mangalore 574 153, India

## Abstract

In the title mol­ecule, C_17_H_14_N_2_OS, the mean plane of the acetamide group forms dihedral angles of 75.79 (5), 81.85 (6) and 12.32 (5)° with the two phenyl rings and the thia­zole ring, respectively. In the crystal, N—H⋯N hydrogen bonds link pairs of mol­ecules into inversion dimers with *R*_2_^2^(8) ring motifs. The crystal packing is further stabilized by C—H⋯π inter­actions and by π–π inter­actions with a centroid–centroid distance of 3.6977 (5) Å.

## Related literature

For the structural similarity of *N*-substituted 2-aryl­acetamides to the lateral chain of natural benzyl­penicillin, see: Mijin & Marinkovic (2006 ▶); Mijin *et al.* (2008 ▶). For the coordination abilities of amides, see: Wu *et al.* (2008 ▶,2010 ▶). For hydrogen-bond motifs, see: Bernstein *et al.* (1995 ▶). For related structures, see: Praveen *et al.* (2011*a* ▶,*b* ▶,*c* ▶); Fun *et al.* (2011*a* ▶,*b* ▶). For standard bond-length data, see: Allen *et al.* (1987 ▶). For the stability of the temperature controller used in the data collection, see: Cosier & Glazer (1986 ▶).
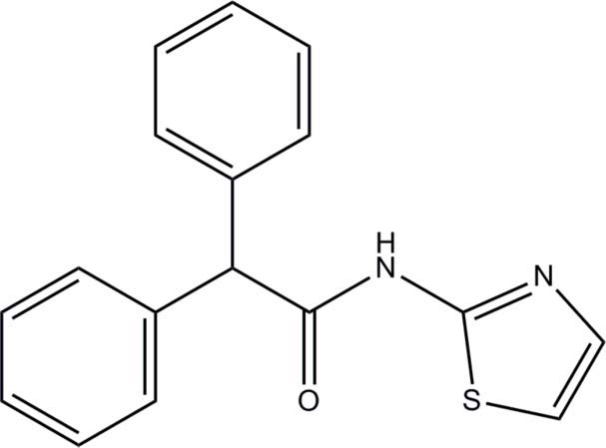


## Experimental

### 

#### Crystal data

C_17_H_14_N_2_OS
*M* *_r_* = 294.36
Monoclinic, 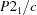

*a* = 5.6915 (1) Å
*b* = 15.1889 (2) Å
*c* = 16.5967 (2) Å
β = 97.845 (1)°
*V* = 1421.32 (4) Å^3^
*Z* = 4
Mo *K*α radiation
μ = 0.23 mm^−1^
*T* = 100 K
0.41 × 0.22 × 0.15 mm

#### Data collection

Bruker SMART APEXII CCD area-detector diffractometer
Absorption correction: multi-scan (*SADABS*; Bruker, 2009 ▶) *T*_min_ = 0.912, *T*_max_ = 0.967
23915 measured reflections
6275 independent reflections
5255 reflections with *I* > 2σ(*I*)
*R*_int_ = 0.026

#### Refinement

*R*[*F* ^2^ > 2σ(*F* ^2^)] = 0.042
*wR*(*F* ^2^) = 0.106
*S* = 1.05
6275 reflections
194 parameters
H atoms treated by a mixture of independent and constrained refinement
Δρ_max_ = 0.50 e Å^−3^
Δρ_min_ = −0.28 e Å^−3^

### 

Data collection: *APEX2* (Bruker, 2009 ▶); cell refinement: *SAINT* (Bruker, 2009 ▶); data reduction: *SAINT*; program(s) used to solve structure: *SHELXTL* (Sheldrick, 2008 ▶); program(s) used to refine structure: *SHELXTL*; molecular graphics: *SHELXTL*; software used to prepare material for publication: *SHELXTL* and *PLATON* (Spek, 2009 ▶).

## Supplementary Material

Crystal structure: contains datablock(s) global, I. DOI: 10.1107/S1600536812013840/lh5446sup1.cif

Structure factors: contains datablock(s) I. DOI: 10.1107/S1600536812013840/lh5446Isup2.hkl

Supplementary material file. DOI: 10.1107/S1600536812013840/lh5446Isup3.cml

Additional supplementary materials:  crystallographic information; 3D view; checkCIF report

## Figures and Tables

**Table 1:** Hydrogen-bond geometry (Å, °) *Cg*1 and *Cg*2 are the centroids of the C1–C6 and C8–C13 rings, respectively.

*D*—H⋯*A*	*D*—H	H⋯*A*	*D*⋯*A*	*D*—H⋯*A*
N1—H1*N*1⋯N2^i^	0.848 (17)	2.116 (17)	2.9600 (12)	173.0 (17)
C1—H1*A*⋯*Cg*2^ii^	0.95	2.88	3.6647 (11)	141
C12—H12*A*⋯*Cg*1^iii^	0.95	2.92	3.6143 (13)	131
C17—H17*A*⋯*Cg*1^iv^	0.95	2.61	3.4381 (11)	146

Symmetry codes: (i) 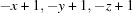; (ii) 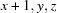; (iii) 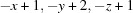; (iv) 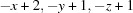.

**Fig. 1.**
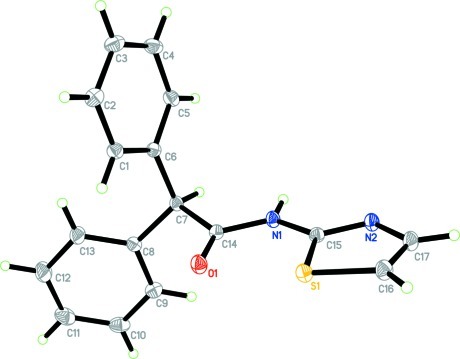
The molecular structure of the title compound, showing 50% probability displacement ellipsoids.

**Fig. 2.**
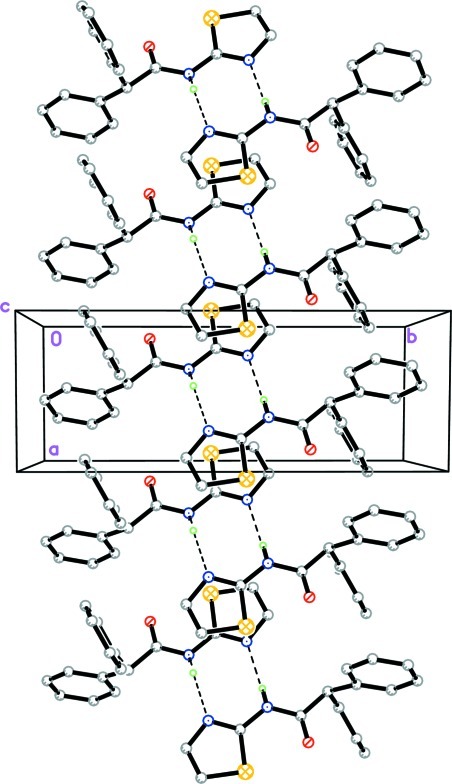
The crystal packing of the title compound, viewed along the c axis. H atoms not involved in the intermolecular interactions (dashed lines) have been omitted for clarity.

## supplementary crystallographic information

### Comment

*N*-Substituted 2-arylacetamides are very interesting compounds because of their structural similarity to the lateral chain of natural benzylpenicillin (Mijin *et al.*, 2006;2008). Amides are also used as ligands due to their excellent coordination abilities (Wu *et al.*, 2008;2010). Crystal structures of some acetamide derivatives *viz*., *N*-(4-Chloro-1,3-benzothiazol-2-yl)-2-(3-methylphenyl) acetamide monohydrate, *N*-(3-Chloro-4-fluorophenyl)-2,2-diphenylacetamide and *N*-(3-Chloro-4-fluorophenyl)-2-(naphthalen-1-yl)acetamide (Praveen *et al.*, 2011*a*,*b*,*c*) have been reported. In continuation of our work on synthesis of amides (Fun *et al.*, 2011 *a*, *b*), we report herein the crystal structure of the title compound (I).

In the title compound (Fig. 1), the mean plane of acetamide (O1/N1/C7/C14) group makes dihedral angles of 75.79 (5)°, 81.85 (6)° and 12.32 (5)° with the two terminal phenyl rings (C1–C6 & C8–C13) and thiazole (S1/N2/C15–C17) ring, respectively. The bond lengths (Allen *et al.*, 1987) and angles are within normal ranges and are comparable to the related structure (Praveen *et al.*, 2011*a*,*b*,*c*; Fun *et al.*, 2011*a*,*b*).

In the crystal (Fig. 2), intermolecular N1—H1N1···N26^i^ hydrogen bonds (Table 1) link molecules to form *R*_2_^2^ (8) ring motifs (Bernstein *et al.*, 1995), leading to the formation of dimers. The crystal packing is further stabilized by C—H···π interactions, involving the C1–C6 ring (centroid *Cg*1) and C8–C13 ring (centroid *Cg*2). Weak π–π interactions are observed with *Cg*3···*Cg*3 = 3.6977 (5) Å [symmetry code: 2-*x*, 1-y, 1-*z*], where *Cg*3 is the centroid of thiazole ring (S1/N2/C15–C17).

### Experimental

Diphenylacetic acid (0.212 g, 1 mmol), 2-amino thiazole (0.1 g, 1 mmol), and 1-ethyl-3-(3-dimethylaminopropyl)-carbodiimide hydrochloride (1.0 g, 0.01 mol) were dissolved in dichloromethane (20 mL). The mixture was stirred in presence of triethylamine at 273 K for about 3 h. The contents were poured into 100 ml of ice-cold aqueous hydrochloric acid with stirring. The resultant mixture was then extracted three times with dichloromethane. The organic layer was washed with saturated NaHCO_3_ solution and brine solution, dried and concentrated under reduced pressure to give the title compound (I). Single crystals were grown from methylene chloride and methanol (1:1) mixture by the slow evaporation method. (*M.P.*: 409–411 K).

### Refinement

Atom H1N1 was located from the difference map and refined freely [N–H = 0.847 (17) Å]. The remaining H atoms were positioned geometrically and refined using a riding model with *U*_iso_(H) = 1.2 *U*_eq_(C) (C—H = 0.95 and 1.00Å). In the final refinement, 7 outliers (-2 21 6), (-2 23 7), (-1 21 7), (0 23 9), (3 19 10), (1 21 9) and (8 0 6) were omitted.

### Crystal data

**Table:**

C_17_H_14_N_2_OS	*F*(000) = 616
*M**_r_* = 294.36	*D*_x_ = 1.376 Mg m^−^^3^
Monoclinic, *P*2_1_/*c*	Mo *K*α radiation, λ = 0.71073 Å
Hall symbol: -P 2ybc	Cell parameters from 9903 reflections
*a* = 5.6915 (1) Å	θ = 2.5–35.1°
*b* = 15.1889 (2) Å	µ = 0.23 mm^−^^1^
*c* = 16.5967 (2) Å	*T* = 100 K
β = 97.845 (1)°	Block, colourless
*V* = 1421.32 (4) Å^3^	0.41 × 0.22 × 0.15 mm
*Z* = 4	

### Data collection

**Table:**

Bruker SMART APEXII CCD area-detector diffractometer	6275 independent reflections
Radiation source: fine-focus sealed tube	5255 reflections with *I* > 2σ(*I*)
Graphite monochromator	*R*_int_ = 0.026
φ and ω scans	θ_max_ = 35.2°, θ_min_ = 2.5°
Absorption correction: multi-scan (*SADABS*; Bruker, 2009)	*h* = −9→9
*T*_min_ = 0.912, *T*_max_ = 0.967	*k* = −16→24
23915 measured reflections	*l* = −20→26

### Refinement

**Table:**

Refinement on *F*^2^	Primary atom site location: structure-invariant direct methods
Least-squares matrix: full	Secondary atom site location: difference Fourier map
*R*[*F*^2^ > 2σ(*F*^2^)] = 0.042	Hydrogen site location: inferred from neighbouring sites
*wR*(*F*^2^) = 0.106	H atoms treated by a mixture of independent and constrained refinement
*S* = 1.05	*w* = 1/[σ^2^(*F*_o_^2^) + (0.0448*P*)^2^ + 0.6018*P*] where *P* = (*F*_o_^2^ + 2*F*_c_^2^)/3
6275 reflections	(Δ/σ)_max_ = 0.002
194 parameters	Δρ_max_ = 0.50 e Å^−^^3^
0 restraints	Δρ_min_ = −0.28 e Å^−^^3^

### Special details

**Table:**

Experimental. The crystal was placed in the cold stream of an Oxford Cryosystems Cobra open-flow nitrogen cryostat (Cosier & Glazer, 1986) operating at 100.0 (1) K.
Geometry. All e.s.d.'s (except the e.s.d. in the dihedral angle between two l.s. planes) are estimated using the full covariance matrix. The cell e.s.d.'s are taken into account individually in the estimation of e.s.d.'s in distances, angles and torsion angles; correlations between e.s.d.'s in cell parameters are only used when they are defined by crystal symmetry. An approximate (isotropic) treatment of cell e.s.d.'s is used for estimating e.s.d.'s involving l.s. planes.
Refinement. Refinement of *F*^2^ against ALL reflections. The weighted *R*-factor *wR* and goodness of fit *S* are based on *F*^2^, conventional *R*-factors *R* are based on *F*, with *F* set to zero for negative *F*^2^. The threshold expression of *F*^2^ > σ(*F*^2^) is used only for calculating *R*-factors(gt) *etc*. and is not relevant to the choice of reflections for refinement. *R*-factors based on *F*^2^ are statistically about twice as large as those based on *F*, and *R*- factors based on ALL data will be even larger.

### Fractional atomic coordinates and isotropic or equivalent isotropic displacement parameters (Å^2^)

**Table:**

	*x*	*y*	*z*	*U*_iso_*/*U*_eq_	
S1	1.07647 (4)	0.542027 (16)	0.646585 (15)	0.01478 (6)	
O1	0.87987 (13)	0.70144 (5)	0.63542 (5)	0.01719 (14)	
N1	0.66503 (15)	0.60037 (5)	0.55657 (5)	0.01345 (15)	
N2	0.75899 (15)	0.44947 (5)	0.55803 (5)	0.01470 (15)	
C1	0.91770 (17)	0.83556 (6)	0.48940 (6)	0.01471 (17)	
H1A	0.9756	0.8479	0.5447	0.018*	
C2	1.04707 (18)	0.86252 (7)	0.42827 (6)	0.01756 (18)	
H2A	1.1911	0.8941	0.4420	0.021*	
C3	0.96610 (19)	0.84336 (7)	0.34702 (6)	0.01904 (19)	
H3A	1.0555	0.8612	0.3055	0.023*	
C4	0.75423 (19)	0.79806 (7)	0.32711 (6)	0.01852 (19)	
H4A	0.6989	0.7845	0.2719	0.022*	
C5	0.62220 (18)	0.77229 (7)	0.38820 (6)	0.01557 (17)	
H5A	0.4761	0.7421	0.3741	0.019*	
C6	0.70288 (16)	0.79043 (6)	0.46986 (6)	0.01264 (16)	
C7	0.56466 (16)	0.75681 (6)	0.53608 (6)	0.01255 (16)	
H7A	0.4160	0.7287	0.5087	0.015*	
C8	0.49405 (16)	0.82524 (6)	0.59564 (6)	0.01339 (16)	
C9	0.36637 (18)	0.79582 (7)	0.65657 (6)	0.01725 (18)	
H9A	0.3371	0.7346	0.6617	0.021*	
C10	0.2815 (2)	0.85479 (8)	0.70978 (7)	0.0214 (2)	
H10A	0.1937	0.8339	0.7506	0.026*	
C11	0.3251 (2)	0.94425 (8)	0.70328 (7)	0.0244 (2)	
H11A	0.2682	0.9847	0.7398	0.029*	
C12	0.4528 (2)	0.97440 (8)	0.64302 (8)	0.0251 (2)	
H12A	0.4833	1.0356	0.6384	0.030*	
C13	0.5361 (2)	0.91505 (7)	0.58931 (7)	0.01917 (19)	
H13A	0.6222	0.9361	0.5481	0.023*	
C14	0.71613 (16)	0.68473 (6)	0.58191 (6)	0.01301 (16)	
C15	0.81095 (16)	0.53022 (6)	0.58261 (6)	0.01279 (16)	
C16	1.12260 (18)	0.43055 (7)	0.63849 (6)	0.01723 (18)	
H16A	1.2576	0.3998	0.6643	0.021*	
C17	0.93884 (18)	0.39295 (7)	0.59006 (6)	0.01634 (17)	
H17A	0.9337	0.3316	0.5788	0.020*	
H1N1	0.548 (3)	0.5890 (11)	0.5207 (10)	0.030 (4)*	

### Atomic displacement parameters (Å^2^)

**Table:**

	*U*^11^	*U*^22^	*U*^33^	*U*^12^	*U*^13^	*U*^23^
S1	0.01364 (10)	0.01391 (11)	0.01602 (11)	0.00093 (7)	−0.00075 (8)	−0.00122 (8)
O1	0.0178 (3)	0.0141 (3)	0.0181 (3)	0.0002 (2)	−0.0030 (3)	−0.0008 (3)
N1	0.0134 (3)	0.0112 (3)	0.0150 (4)	0.0011 (3)	−0.0009 (3)	−0.0007 (3)
N2	0.0149 (3)	0.0119 (3)	0.0168 (4)	0.0012 (3)	0.0004 (3)	−0.0009 (3)
C1	0.0146 (4)	0.0153 (4)	0.0138 (4)	0.0009 (3)	0.0005 (3)	0.0003 (3)
C2	0.0151 (4)	0.0185 (4)	0.0192 (4)	0.0009 (3)	0.0031 (3)	0.0023 (4)
C3	0.0205 (4)	0.0208 (5)	0.0166 (4)	0.0049 (4)	0.0054 (3)	0.0046 (4)
C4	0.0225 (5)	0.0201 (5)	0.0128 (4)	0.0045 (4)	0.0014 (3)	0.0000 (3)
C5	0.0161 (4)	0.0156 (4)	0.0142 (4)	0.0026 (3)	−0.0007 (3)	−0.0015 (3)
C6	0.0134 (4)	0.0118 (4)	0.0127 (4)	0.0024 (3)	0.0014 (3)	0.0003 (3)
C7	0.0126 (4)	0.0114 (4)	0.0133 (4)	0.0008 (3)	0.0008 (3)	−0.0002 (3)
C8	0.0126 (4)	0.0139 (4)	0.0135 (4)	0.0021 (3)	0.0009 (3)	−0.0009 (3)
C9	0.0156 (4)	0.0192 (4)	0.0172 (4)	0.0015 (3)	0.0032 (3)	0.0018 (3)
C10	0.0201 (5)	0.0283 (5)	0.0166 (4)	0.0051 (4)	0.0057 (4)	0.0008 (4)
C11	0.0271 (5)	0.0261 (5)	0.0207 (5)	0.0083 (4)	0.0061 (4)	−0.0047 (4)
C12	0.0321 (6)	0.0159 (5)	0.0289 (6)	0.0043 (4)	0.0101 (5)	−0.0039 (4)
C13	0.0236 (5)	0.0137 (4)	0.0216 (5)	0.0023 (3)	0.0081 (4)	−0.0003 (4)
C14	0.0142 (4)	0.0119 (4)	0.0131 (4)	0.0000 (3)	0.0024 (3)	0.0000 (3)
C15	0.0126 (4)	0.0133 (4)	0.0125 (4)	0.0003 (3)	0.0019 (3)	0.0002 (3)
C16	0.0167 (4)	0.0152 (4)	0.0192 (4)	0.0036 (3)	0.0003 (3)	−0.0002 (3)
C17	0.0166 (4)	0.0129 (4)	0.0193 (4)	0.0031 (3)	0.0013 (3)	−0.0006 (3)

### Geometric parameters (Å, º)

**Table:**

S1—C16	1.7215 (10)	C6—C7	1.5243 (13)
S1—C15	1.7336 (10)	C7—C8	1.5257 (13)
O1—C14	1.2230 (12)	C7—C14	1.5302 (13)
N1—C14	1.3675 (12)	C7—H7A	1.0000
N1—C15	1.3833 (12)	C8—C13	1.3914 (14)
N1—H1N1	0.847 (17)	C8—C9	1.3967 (14)
N2—C15	1.3135 (12)	C9—C10	1.3899 (15)
N2—C17	1.3853 (13)	C9—H9A	0.9500
C1—C2	1.3939 (14)	C10—C11	1.3881 (17)
C1—C6	1.4006 (13)	C10—H10A	0.9500
C1—H1A	0.9500	C11—C12	1.3914 (18)
C2—C3	1.3950 (15)	C11—H11A	0.9500
C2—H2A	0.9500	C12—C13	1.3954 (15)
C3—C4	1.3885 (16)	C12—H12A	0.9500
C3—H3A	0.9500	C13—H13A	0.9500
C4—C5	1.3978 (15)	C16—C17	1.3552 (14)
C4—H4A	0.9500	C16—H16A	0.9500
C5—C6	1.3977 (13)	C17—H17A	0.9500
C5—H5A	0.9500		
			
C16—S1—C15	88.85 (5)	C13—C8—C7	123.77 (9)
C14—N1—C15	122.15 (8)	C9—C8—C7	117.40 (9)
C14—N1—H1N1	121.4 (11)	C10—C9—C8	120.93 (10)
C15—N1—H1N1	116.2 (11)	C10—C9—H9A	119.5
C15—N2—C17	109.65 (8)	C8—C9—H9A	119.5
C2—C1—C6	120.42 (9)	C11—C10—C9	120.01 (10)
C2—C1—H1A	119.8	C11—C10—H10A	120.0
C6—C1—H1A	119.8	C9—C10—H10A	120.0
C1—C2—C3	120.32 (10)	C10—C11—C12	119.63 (10)
C1—C2—H2A	119.8	C10—C11—H11A	120.2
C3—C2—H2A	119.8	C12—C11—H11A	120.2
C4—C3—C2	119.67 (10)	C11—C12—C13	120.18 (11)
C4—C3—H3A	120.2	C11—C12—H12A	119.9
C2—C3—H3A	120.2	C13—C12—H12A	119.9
C3—C4—C5	120.10 (9)	C8—C13—C12	120.55 (10)
C3—C4—H4A	120.0	C8—C13—H13A	119.7
C5—C4—H4A	120.0	C12—C13—H13A	119.7
C6—C5—C4	120.68 (9)	O1—C14—N1	121.79 (9)
C6—C5—H5A	119.7	O1—C14—C7	122.29 (9)
C4—C5—H5A	119.7	N1—C14—C7	115.83 (8)
C5—C6—C1	118.81 (9)	N2—C15—N1	121.47 (9)
C5—C6—C7	119.96 (9)	N2—C15—S1	115.30 (7)
C1—C6—C7	121.14 (8)	N1—C15—S1	123.21 (7)
C6—C7—C8	116.51 (8)	C17—C16—S1	110.32 (7)
C6—C7—C14	106.68 (7)	C17—C16—H16A	124.8
C8—C7—C14	110.22 (8)	S1—C16—H16A	124.8
C6—C7—H7A	107.7	C16—C17—N2	115.87 (9)
C8—C7—H7A	107.7	C16—C17—H17A	122.1
C14—C7—H7A	107.7	N2—C17—H17A	122.1
C13—C8—C9	118.70 (9)		
			
C6—C1—C2—C3	−1.20 (15)	C10—C11—C12—C13	0.13 (19)
C1—C2—C3—C4	0.70 (16)	C9—C8—C13—C12	0.21 (16)
C2—C3—C4—C5	0.39 (16)	C7—C8—C13—C12	175.82 (10)
C3—C4—C5—C6	−1.01 (15)	C11—C12—C13—C8	−0.42 (18)
C4—C5—C6—C1	0.51 (14)	C15—N1—C14—O1	−7.86 (15)
C4—C5—C6—C7	−176.13 (9)	C15—N1—C14—C7	168.96 (8)
C2—C1—C6—C5	0.59 (14)	C6—C7—C14—O1	81.74 (11)
C2—C1—C6—C7	177.19 (9)	C8—C7—C14—O1	−45.64 (12)
C5—C6—C7—C8	−126.30 (9)	C6—C7—C14—N1	−95.07 (9)
C1—C6—C7—C8	57.14 (12)	C8—C7—C14—N1	137.56 (9)
C5—C6—C7—C14	110.15 (9)	C17—N2—C15—N1	177.26 (9)
C1—C6—C7—C14	−66.41 (11)	C17—N2—C15—S1	−0.88 (11)
C6—C7—C8—C13	4.06 (13)	C14—N1—C15—N2	179.68 (9)
C14—C7—C8—C13	125.77 (10)	C14—N1—C15—S1	−2.33 (13)
C6—C7—C8—C9	179.72 (8)	C16—S1—C15—N2	0.84 (8)
C14—C7—C8—C9	−58.57 (11)	C16—S1—C15—N1	−177.26 (9)
C13—C8—C9—C10	0.29 (15)	C15—S1—C16—C17	−0.54 (8)
C7—C8—C9—C10	−175.60 (9)	S1—C16—C17—N2	0.17 (12)
C8—C9—C10—C11	−0.59 (16)	C15—N2—C17—C16	0.44 (13)
C9—C10—C11—C12	0.37 (18)		

### Hydrogen-bond geometry (Å, º)

Cg1 and Cg2 are the centroids of the C1–C6 and C8–C13 rings, respectively.

**Table:**

*D*—H···*A*	*D*—H	H···*A*	*D*···*A*	*D*—H···*A*
N1—H1*N*1···N2^i^	0.848 (17)	2.116 (17)	2.9600 (12)	173.0 (17)
C1—H1*A*···*Cg*2^ii^	0.95	2.88	3.6647 (11)	141
C12—H12*A*···*Cg*1^iii^	0.95	2.92	3.6143 (13)	131
C17—H17*A*···*Cg*1^iv^	0.95	2.61	3.4381 (11)	146

Symmetry codes: (i) −*x*+1, −*y*+1, −*z*+1; (ii) *x*+1, *y*, *z*; (iii) −*x*+1, −*y*+2, −*z*+1; (iv) −*x*+2, −*y*+1, −*z*+1.
